# The Recognition

**Published:** 1882-09

**Authors:** Ad. Lippe

**Affiliations:** Philadelphia


### THE RECOGNITION.

AD. LIPPE, M. D., PHILADELPHIA.

The American Medical Association declined to recognize the delegates of the New York State Medical Society because said New York Society had passed, at its last meeting, a resolution of "Recognition" of all properly-graduated doctors, of course, including the homœopathists. This action was not in harmony with the well-established Code of Ethics of the American Medical Association. The doctors sat in council, and deliberately came to the conclusion that here was a terrible disease afflicting a section of the body medical, and they all cried out for "heroic treatment." The afflicted section had committed an unpardonable sin and must "be removed." These progressively-scientific doctors did for once forget their battle cry, "*tolle causum*," and cried for heroic practice—amputation! There was no deliberate examination of the sick section; nobody asked for the cause of their transgression! If the cause could be found, probably that cause could be removed and the section could be saved. Of course, the New York Society had done a very improper thing in passing that act of "Recognition." How came it to be done? Sheltered behind a high Chinese wall, many centuries old, in fact built ever since the medical men of early history established "a caste" of their own, and over which wall, every intruder, who did not accept the prevailing *opinions* of the caste, was pitched. There stands the High Priests of the caste, carefully watching the high wall, that no intruder might disturb their ancient faith in their perpetual rights as a caste. However frequently the *opinions* of the leaders of the caste changed, they persistently guarded against the introduction of fundamental principles of laws governing the healing art, and, above all, they abhorred the idea that natural laws should become the foundation of the laws that could and would govern the healing art. It happened early in the year 1882, that the New York State Medical Society stood guard over this impregnable wall, and from the other side of the wall, came a sound of trumpets for a "parley!" The sentinels mounted the wall and behold, there came, clothed in sackcloth and ashes, a set of men who carried the homœopathic banner upside down, and they chanted their own conviction, that the progress in science had annihilated Hahnemann's peculiar tenets, and that they, the chanters, had only held on to a distinctive name of an exclusive system of medicine on account of the gains it might bring them; that they were the representatives of the homœopathic school of medicine, and ready to abandon, at once, Hahnemann, his teachings, and the name, provided the sacred and old caste would recognize them. And so they were recognized by the New York Society, then on guard; and these now find themselves not recognized by "The American Medical Association." There are members of the New York Society who have expressed their disapproval of the action of their Society and show signs of rebellion. That the rebellious members of "the caste" may be properly understood, we publish what they have to say, from the New York *Herald*, June 11th, 1882:

### DOCTORS IN COUNCIL.

Prejudices die hard even in the minds of learned men, and this truth, perhaps, scarcely needed at the hands of the American Medical Association the further demonstration supplied by its action in refusing to admit at its annual gathering the delegates of the New York Society, because this body has adopted a liberal and enlightened rule in regard to physicians of the homœopathic school. That somewhat famous personage who did not like Dr. Fell, and who, though at a loss to assign any good reasons for his objections to that particular practitioner, was none the less positive that he did not, could not, should not and would not like the said doctor, has stood for some generations as the type of a person who, through indulging himself in irrational antipathies, has become a victim to them, and finds that the concrete malevolences of his history overrule his intelligence and are the really controlling facts in his life. But he must give place as a type to the American Medical Association, which not only will not tolerate the professional existence of the doctor it does not like, but confounds in a common wrath every one who holds that there is any possible *modus vivendi* with this dreadful fellow Fell.

For a great while the relation in practice of physicians of one or another school has been a delicate subject, and physicians in large cities have felt its difficulty more keenly than those in the rural districts. At one time, when the homœopathic issue first arose, there was no difficulty. It was all settled in one word. Doctors of the old school merely shouted "quackery" at the others, and that was the end of it. Perhaps this was at that time a good enough end, too; for Homœopathy, as then commonly seen here, was a mere application of certain rigid formulæ to symptoms imagined to be the same, and was neither a scientific nor an empirical use of remedial measures. It was taken up by hordes of ingenious persons of that order of humanity which tries its hand at all trades, and seldom succeeds at any, and they went about with pockets full of pellets of the millionth dilution and tables of Hahnemann's "provings," and guessed out the relation of one to another at the bedside in a sort of pitch and toss way, doing, it must be admitted, uncommonly little harm. But, if anybody imagines that that sort of Homœopathy exists here now, he does not know how the world wags. Homœopathic doctors are now as well instructed as the great mass of the other doctors, and there are desirable points of advancement in that particular open to all of them; and, besides this, those extravagances of theory which were the peculiar paraphernalia of the followers of Hahnemann have been dropped by the wayside. Fabulous dilutions like "dammes" seem to have had their day—and there is no more now, we believe, in the theory of similars, or not much more, than every thoroughly instructed physician admits—no more than the physician, instructed or otherwise, acts upon when he uses the vaccine poison to prevent variola.

In the light of this change we may see the nature of the problem presented to the practitioners of to-day. However it may be in remote rural regions, it is certain that the competitions of profitable practice in cities like this have had their effect in Homœopathy as in other schools. He would be a very hardy medical bumpkin of the cross-roads who should stand up in a medical assembly to denounce as capable of quackery the recently deceased Dr. Gray. Our doctors have to meet such men in consultation, or they have to give to the public a reason why. They cannot afford to give a ridiculous reason. They naturally do not want to tell the friends of a patient that they care more for consistency in respect to a thin theory than for curing disease or saving life. For all these reasons, the doctors of this State, observing that the tangible objections to consultations with homœopathists no longer exist, declared that fact. They were right, and they could not honestly do otherwise. They, moreover, were under an obligation to do it. Our county medical societies are, under our State laws, authorities on the qualifications of practitioners. They have a right to pass upon a man's qualifications. They can judge of his knowledge, but not of his opinions; but, having declared that by knowledge he is entitled to practice, how can they then refuse to consult with him because of his opinions?

But it will not hurt the New York State Medical Society to be under the ban of the National Society, and we hope it may not repent of its temerity so far as to abandon the liberal and proper position it has taken. There is, however, one topic in this connection that may be regarded as of high and heroic interest. Now that our regular medical organizations and practitioners are martyrs for the right, and know how it is themselves, will they take any action with regard to those enlightened practitioners who long since looked liberally on the homœopathic practice, and whom our societies have persecuted for years for that very offense for which the societies are persecuted now?

*Comments:* The author or authors of this paper, are utterly ignorant of the history of Homœopathy, and of its exclusive tenets. The frauds who approached the New York sentinels, were not representative men of the homœopathic school; they were men who had had the effrontery to assume a name to which they were never entitled, they NEVER even attempted to learn Homœopathy, and concluded to abuse a healing art they could not practice. The most painstaking parts of the practice they abandoned—instead of learning how to examine every sick person carefully, and just as carefully find the similar remedy in the homœopathic materia medica, they lazily diagnosticated a form of disease, and if, as usual, the remedy had been carelessly chosen, and more absurdly administered without success, then these lazy and ignorant pretenders at once resort to the common palliative treatment of the old school. And even worse yet, they declare that all men who practice Homœopathy, are just the detestable frauds as they showed themselves to be. Under the belief that these men were representative men, the recognition was granted: of course it was a hasty act, and a thoughtless one. After the American Medical Association has more fully examined into the case, it will become apparent that peace can be restored, after the New York Society has been shown how they were deceived. Consultations between homœopathists and allopathists will not be asked for by any intelligent person; they must end in a surrender by one or the other physician; they may agree as to a "diagnosis," but they can never possibly agree on the remedial means (therapeutics) to be used for the restoration of health; is it not obvious that the sick, for whose benefit this consultation has been called, cannot be benefited in the least? It is not "*rational*" to suppose that two systems of medicine, diametrically opposed one to another, can beneficially (for the sick) be applied jointly! The American Medical Association will be able, readily, to convince the rash, erring brethren of New York that they have been sadly deceived by a desperate set of "*Deserters.*"

